# Spillover effects of public school integration in the southern United States: diverging trends in statewide annual breast cancer incidence, 2001–2019

**DOI:** 10.1007/s10552-025-02124-x

**Published:** 2026-02-06

**Authors:** Mohin Chanpura

**Affiliations:** Department of Pharmaceutical and Health Economics, Alfred E. Mann School of Pharmacy and Pharmaceutical Sciences, University of Southern California, Los Angeles, CA USA

**Keywords:** Black Americans, Breast cancer, Education, Health disparities, Jim Crow, School segregation

## Abstract

**Purpose:**

Education has long been established as a key determinant of health, with prior research linking higher educational attainment to improved health outcomes and better self-management of chronic disease. This analysis investigated diverging trends in breast cancer incidence among cohorts of non-Hispanic Black (NHB) and non-Hispanic White (NHW) women of school age during public school integration in the southern United States following *Brown v. Board of Education*.

**Methods:**

Using U.S. Cancer Statistics data from 2001 to 2019, I conducted several segmented linear regressions comparing trends in annual breast cancer incidence among women in Missouri and South Carolina born between 1937 and 1950, as these two states’ responses to *Brown v. Board* starkly contrasted one another. I hypothesized that, given Missouri’s swift integration of public schools in 1955—versus South Carolina’s continued resistance to integration through 1970—I’d observe significant changes in trend after age 62, the median age of breast cancer diagnosis, among NHB women in Missouri born from 1944 to 1950.

**Results:**

Among NHB women in Missouri born from 1944 to 1950, I observed a significant reduction in breast cancer incidence post-cutoff relative to NHW women in Missouri (*p* < 0.001) and NHB women in South Carolina (*p* = 0.017) within the same birth cohort. In both states, I observed no significant differences in incidence among NHB and NHW women born from 1937 to 1943.

**Conclusion:**

Public school integration appears to have been associated with long-term protective spillover effects against breast cancer among younger Black students in Missouri, underscoring the importance of, and continued need for, policies that combat lingering de facto school segregation.

## Introduction

Racial segregation in the United States of America is well documented as having had a profound impact on disparities in cancer outcomes between non-Hispanic Black and White Americans. Per Jatoi et al., age-adjusted breast cancer mortality in the U.S. is approximately 40% higher among non-Hispanic Black women than non-Hispanic White women, despite lower incidence among the former [1]. Residing in racially segregated neighborhoods—often due to discriminatory practices like redlining rather than personal choice—is associated with an increased likelihood of late-stage diagnosis of, and therefore mortality from, breast and lung cancers [2]. Zeroing in on the effects of historical *de jure* segregation in the USA on breast cancer outcomes, Krieger et al. find that while non-Hispanic Black women are at significantly greater risk of developing estrogen receptor-negative (ER-) breast cancer than non-Hispanic White women overall, this disparity is amplified for women born in the southern United States before 1965 under the rule of “Jim Crow” laws mandating racial segregation in all public facilities and places of business [3].

While the broader effects of racial segregation on cancer outcomes have been well established, the specific pathways through which educational segregation—particularly in primary and secondary schools—may influence long-term cancer risk remain underexplored. Under the Grossman model of health demand, education is identified as the single most important determinant of how efficiently an individual can increase their stock of health by consuming one additional unit of medical care [4]. Ehrlich expands on this relationship using optimal control theory to model significant interactions between educational capital and health capital over one’s lifespan [5]. Goldman and Smith further demonstrate this mechanism, showing that the better educated an individual is, the better their self-management of disease—a finding they confirm in a large cohort of HIV patients being treated with highly active antiretroviral therapy (HAART) [6].

For almost a full century prior to *Brown v. Board of Education*, the landmark U.S. Supreme Court case that unanimously ruled the segregation of White and Black public school students on the basis of race unconstitutional in 1954, racial minorities in 20 southern states had lived under the rule of discriminatory Jim Crow laws [3, 7]. The separate public schools that were established for Black students during this era were anything but equal, as state governments divested a disproportionate amount of public money into schools for White children. Many schools for Black students had leaking roofs, sagging floors, and windows without glass, and faced shortages of desks, books, and qualified teachers. Additionally, states imposed limits on what Black students could be taught, resulting in limited literacy relative to White students with equivalent years of schooling [8].

As of May 17, 1954, the day *Brown v. Board* was decided, 17 southern states still mandated public school segregation. At the start of the 1955–1956 school year, each of these states varied widely in efforts made to comply with the Supreme Court’s order to enforce public school integration with “deliberate speed.” Per *Time* magazine, which issued a report card of the states’ progresses toward school desegregation, grading each state from “A” to “F,” Missouri (A) was the least resistant to the court’s order, having integrated 80% of its Black students into previously white-only schools starting September 1955. By contrast, while South Carolina (F) was one of several states that made no efforts to desegregate, it was the only state to pass legislation overtly threatening to cut funding from any school district permitting integration [9].

Given that the Grossman model of health demand establishes a clear relationship between an individual’s level of education and their future health status and that Krieger et al. show significant associations between being born in the Jim Crow era South and an increased risk of breast cancer among non-Hispanic Black women relative to non-Hispanic White women, I performed several segmented linear regressions on trends in annual breast cancer incidence rates among multiple birth cohorts in Missouri and South Carolina to explore potential associations between the integration of public schools in Missouri starting September 1955 and altered trends in breast cancer incidence among non-Hispanic Black women relative to non-Hispanic White women [3, 5]. Evidence of school integration reducing disparities in breast cancer incidence between non-Hispanic Black and White women would substantially enhance policymakers’ knowledge of how interventions aimed at combating de facto public school segregation, still rampant in the U.S. today, could further translate to reduced racial inequity in both cancer and overall health outcomes.

## Methods

### Data source

To conduct this analysis, I first obtained access to United States Cancer Statistics (USCS) data spanning 2001–2021. USCS compiles cancer incidence data reported to the Centers for Disease Control and Prevention’s (CDC’s) National Program of Cancer Registries (NPCR) and the National Cancer Institute’s (NCI’s) Surveillance, Epidemiology, and End Results (SEER) Program into a single public use database with cancer incidence data for all 50 states, Washington, D.C., and Puerto Rico [10]. The database was analyzed using NCI’s SEER*Stat 8.4.5 software package. I restricted the analytic dataset to statewide breast cancer incidence among non-Hispanic Black and non-Hispanic White women born between 1937 and 1950, as this cohort would have been of school age when Missouri integrated its public schools in September 1955 [9].

### Exposure variables: home state and year of birth

Given Missouri’s swift integration of public schools following *Brown v. Board* relative to 16 other Jim Crow states, and South Carolina’s continued resistance to public school integration through 1970, I chose to focus this analysis specifically on breast cancer incidence among female residents of these two states, hypothesizing that any significant changes in annual incidence rates observed among non-Hispanic Black women in Missouri would not be simultaneously observed among non-Hispanic Black women in South Carolina [9]. It should be noted that, while USCS only allows researchers to filter cancer incidence data by home state at diagnosis, not by state of birth, the Federal Reserve finds that both Missouri and South Carolina are relatively sticky with respect to their retention of native residents. As of 2021, 68% of all Missouri residents and 72% of all South Carolina residents were born in their respective home states [11].

As Sanbonmatsu, Kling, and others note that the benefits of policy interventions that move underprivileged students to better schools are primarily observed in younger children (i.e., ages 6–10) with less lifetime exposure to poverty than teenagers, I further stratified the analytic dataset into two birth cohorts: those born from 1937 to 1943 (11–18 years of age at the time of Missouri public school integration), and those born from 1944 to 1950 (4–11 years of age at the time of integration) [12]. I hypothesized that, given this assumption, significant changes in annual incidence rates would only be observed among non-Hispanic Black women in Missouri born from 1944 to 1950, and not those born from 1937 to 1943.

### Outcome: breast cancer incidence rates (per 100,000) over time

I used SEER’s AYA Site Recode 2020 Revision variable to identify incident cases of breast cancer (defined as any of the following: 9.6 carcinoma of breast, 9.6.1 breast—infiltrating duct, 9.6.2 breast—adenocarcinoma, 9.6.3 breast—lobular, 9.6.4 breast—phyllodes, 9.6.5 breast—medullary, 9.6.6 breast—paget, 9.6.7 breast—ductal, 9.6.8 breast—metaplastic, 9.6.9 breast—inflammatory, 9.6.10 breast—other) from 2001 to 2019 and used McNeil et al.’s method of cubic spline interpolation to estimate total annual birth cohort populations using 2000, 2010, and 2020 U.S. Census data sourced from the IPUMS National Historical Geographic Information System (NHGIS) [13]. I then derived annual incidence rates per 100,000 from 2001 to 2019 for each cohort of interest using the reported numbers of cases and interpolated population estimates.

Given that the median age of breast cancer diagnosis in the U.S. is 61–62 years and that cancer risk increases exponentially as individuals’ age into their 60s and 70s, I hypothesized that the treatment effect would first be observable at a median age of 62 years [14, 15]. I therefore set the cutoff points for segmented linear regression at 62, anticipating significant differences in trends in annual breast cancer incidence from median ages 62 onward, and non-significant differences in trend between median ages 54–62.

### Covariates

In order to minimize the capture of incidence data from women not born and raised in the Jim Crow era South, I restricted the analytic dataset to incidence among non-Hispanic Black and non-Hispanic White women using SEER’s race and origin recode variable, which classifies each case as either NHW (non-Hispanic White), NHB (non-Hispanic Black), NHAIAN (non-Hispanic American Indian/Alaska Native), NHAPI (non-Hispanic Asian/Pacific Islander), or Hispanic. I hypothesized that any changes in trend observed among non-Hispanic Black women in Missouri born between 1944 and 1950 would not be observed among non-Hispanic White women of the same age if the effect was truly correlated with integrating Black students into formerly white-only public schools. As a robustness check, I referenced data from the CDC’s Behavioral Risk Factor Surveillance System (BRFSS) survey to estimate changes in breast cancer screening rates among the 1944–1950 birth cohort from 2008 (then 57–64 years of age) to 2016 (then 65–72 years of age), with screening defined as having had a mammogram within the past two years [16, 17].

### Statistical analysis

Using Stata® 14.0, I performed several segmented linear regressions on annual incidence rates by median age to compare incidence among non-Hispanic Black and White women in Missouri and South Carolina born from 1944 to 1950 and among corresponding cohorts born from 1937 to 1943. I used Wald tests to assess whether growth rates of annual breast cancer incidence past the median age of 62 were significantly lower in non-Hispanic Black women in Missouri than in non-Hispanic White women in Missouri and non-Hispanic Black women in South Carolina within each birth cohort, and to assess whether growth rates past the median age of 69 were significantly lower among non-Hispanic Black women in Missouri than in non-Hispanic White women in Missouri and non-Hispanic Black women in South Carolina born from 1937 to 1943—in order to test for potential period effects contemporaneously affecting both cohorts around a 2009 cutoff [18]. QueryI additionally built two generalized linear models (GLMs) with a gamma family and log link in order to perform sensitivity and robustness checks and again employed Wald tests to assess whether predicted annual percentage growth in incidence per 100,000 was significantly lower in non-Hispanic Black women in Missouri than in their non-Hispanic White peers in Missouri or their non-Hispanic Black peers in South Carolina. Unlike the segmented linear regression models, which estimated absolute year-over-year change in incidence rates, these GLMs yielded multiplicative effects interpretable as percent change in incidence per each additional year of age.

## Results

As noted in Table 1, I identified a total of 15,284 incident cases of breast cancer from 2001 to 2019 among Missouri women born between 1944 and 1950 (1,482 among non-Hispanic Black women, 13,802 among non-Hispanic White women), and a total of 12,815 incident cases over the same timespan among Missouri women born between 1937 and 1943 (982 among non-Hispanic Black women, 11,833 among non-Hispanic White women). I additionally identified a total of 13,726 incident cases of breast cancer among South Carolina women born between 1944 and 1950 (3,007 among non-Hispanic Black women, 10,719 among non-Hispanic White women), and a total of 10,132 cases among South Carolina women born between 1937 and 1943 (1,994 among non-Hispanic Black women, 8,138 among non-Hispanic White women).

**Table 1:** Annual incident cases of breast cancer by state, birth cohort, and race (2001–2019)

*State*	Missouri (MO)				South Carolina (SC)			
*Cohort*	1944–1950 Births		1937–1943 Births		1944–1950 Births		1937–1943 Births	
*Race*	Black	White	Black	White	Black	White	Black	White
2001	61	546	57	621	123	343	82	365
2002	74	582	44	597	100	365	92	374
2003	48	554	43	598	125	359	96	338
2004	62	525	48	572	128	418	103	352
2005	70	548	48	602	129	432	91	350
2006	66	595	57	582	125	457	102	412
2007	74	592	46	588	139	483	133	425
2008	75	690	55	648	174	498	101	459
2009	97	747	60	635	181	515	129	414
2010	79	727	49	612	150	662	94	439
2011	99	743	65	684	163	651	107	483
2012	87	794	57	663	168	665	130	496
2013	86	839	57	668	166	734	102	465
2014	95	862	42	697	163	746	112	462
2015	91	874	67	647	189	679	116	500
2016	85	894	48	630	211	695	99	432
2017	97	892	64	607	201	643	109	478
2018	62	861	37	594	190	679	92	459
2019	74	917	38	588	182	695	103	435

**Fig. 1:**
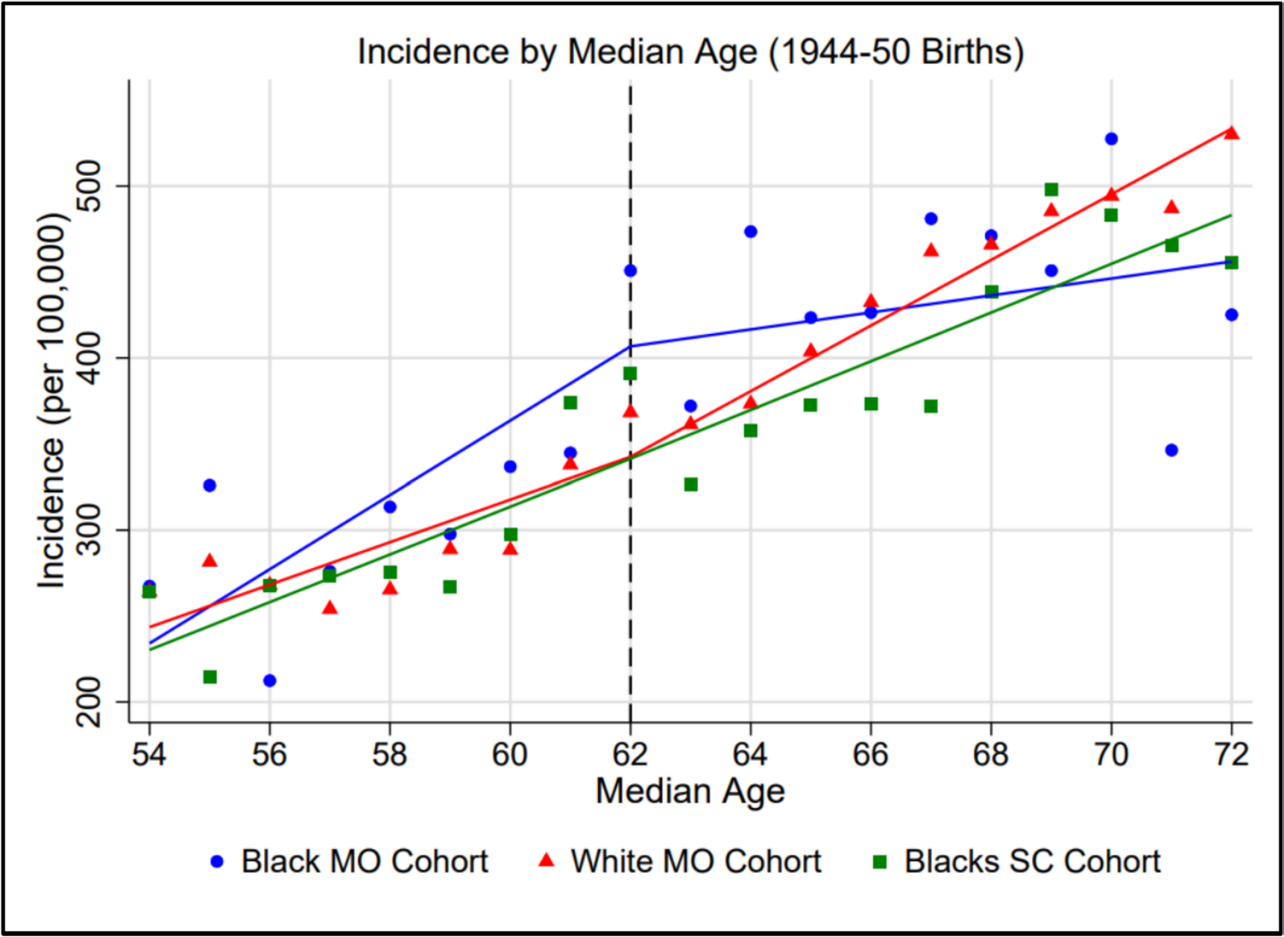
Breast cancer incidence rates per 100,000 by median age (1944–1950 births). Data points represent observed incidence rates per 100,000 while lines of best fit represent regressionestimated predicted incidence rates

As shown in Fig. 1, I observed markedly higher growth in annual breast cancer incidence rates among non-Hispanic Black Missouri women from the median ages of 54–62 than in their non-Hispanic White counterparts in Missouri (*p* = 0.049) and non-Hispanic Black counterparts in South Carolina (*p* = 0.083) born between 1944 and 1950. However, as predicted, I observed significantly decreased growth in annual incidence rates among non-Hispanic Black Missouri women past the median age of 62 while observing increased growth among both comparator groups across the post-cutoff period. Table 2 lists annual incidence rates per 100,000 by state, birth cohort, and race.

**Table 2:** Annual breast cancer incidence rates (per 100,000) by state, birth cohort, and race

*State*	Missouri (MO)				South Carolina (SC)			
*Cohort*	1944–1950 Births		1937–1943 Births		1944–1950 Births		1937–1943 Births	
*Race*	Black	White	Black	White	Black	White	Black	White
2001	267.4	264.2	357.1	390.7	264.0	262.4	269.4	364.3
2002	325.9	281.5	279.0	377.7	214.3	275.3	299.4	370.7
2003	212.4	268.0	276.0	380.6	267.5	267.0	313.7	332.8
2004	276.0	254.1	312.0	365.5	273.4	307.1	337.7	344.8
2005	313.5	265.4	316.5	388.8	275.6	313.4	300.0	341.6
2006	297.7	288.9	381.7	379.4	267.3	327.9	338.9	401.2
2007	336.7	288.4	313.1	387.2	297.6	343.1	445.7	413.7
2008	344.7	337.9	381.6	432.2	373.7	350.7	342.4	447.5
2009	450.8	368.3	425.1	429.8	390.9	360.1	443.4	405.7
2010	372.0	361.3	355.4	421.2	326.2	460.1	328.7	433.6
2011	473.5	373.3	483.9	480.4	357.7	450.7	382.3	482.6
2012	423.4	403.7	437.1	476.6	372.7	458.9	475.6	502.8
2013	426.4	432.4	451.5	492.5	373.0	505.8	383.9	480.0
2014	481.0	461.9	344.7	528.8	371.8	514.0	434.4	486.9
2015	471.1	465.9	571.1	506.2	438.3	468.0	465.7	539.4
2016	450.8	485.3	426.2	509.6	497.7	479.9	412.7	478.3
2017	527.5	494.3	593.8	509.0	482.9	445.1	473.3	544.4
2018	346.3	487.0	360.0	517.4	465.7	471.3	417.5	538.8
2019	425.1	530.0	388.7	533.1	455.3	483.7	489.5	527.1

Wald tests revealed that, among women born between 1944 and 1950, the growth of annual breast cancer incidence rates per 100,000 was significantly lower among non-Hispanic Black women in Missouri relative to both non-Hispanic White women in Missouri (*p* < 0.001) and non-Hispanic Black women in South Carolina (*p* = 0.017) across the post-cutoff period. Table 3 lists estimated growth rates (with 95% confidence intervals) across both the pre- and post-cutoff periods in all three groups.

**Table 3:** Pre- and post-cutoff growth in annual incidence rate per 100,000 (1944–1950 births)

		Pre-cutoff (Median Ages 54–62)			Post-cutoff (Median Ages 62–72)		
State	Race	Δ (Slope)	95% Low	95% High	Δ (Slope)	95% Low	95% High
MO	Black	+21.57	+13.81	+29.33	+4.94	-1.08	+10.96
MO	White	+12.36	+4.61	+20.12	+19.10	+13.08	+25.11
SC	Black	+13.87	+6.12	+21.63	+14.18	+8.16	+20.19

**Fig. 2:**
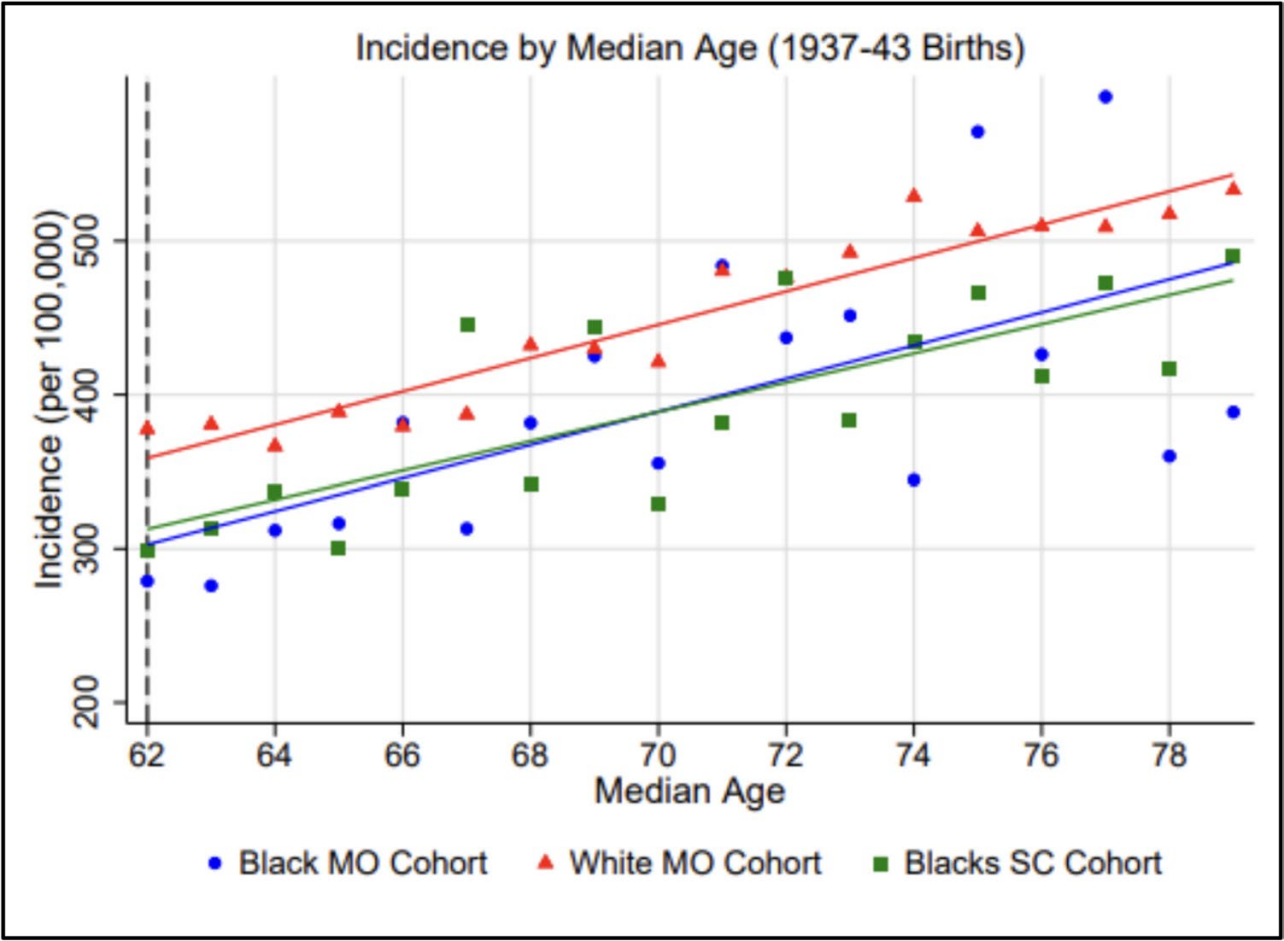
Breast cancer incidence rates per 100,00 by median age (1937–1943 births). Data points represent observed incidence rates per 100,000 while lines of best fit represent regressionestimated predicted incidence rates

As shown in Fig. 2, I observed parallel growth in annual breast cancer incidence rates after the median age of 62 among non-Hispanic Black and White women in Missouri and non-Hispanic Black women in South Carolina born from 1937 to 1943, supporting the hypothesis that reduced incidence would only be observed among the younger birth cohort. Wald testing confirmed that the post-cutoff growth rate of annual incidence per 100,000 was not significantly lower among non-Hispanic Black women in Missouri than among either non-Hispanic White women in Missouri (*p* = 0.488) or non-Hispanic Black women in South Carolina (*p* = 0.298).

Wald testing additionally confirmed that the growth rate of annual incidence per 100,000 past the median age of 69 was not significantly lower among non-Hispanic Black women in Missouri than among either non-Hispanic White women in Missouri (*p* = 0.089) or non-Hispanic Black women in South Carolina (*p* = 0.375) born from 1937 to 1943, implying that potential period effects occurring around a 2009 cutoff did not significantly alter annual incidence among this birth cohort. Table 4 lists estimated growth rates (with 95% confidence intervals) across the post-cutoff period in all three groups.

**Table 4:** Post-cutoff growth in annual incidence rate per 100,000 (1937–1943 births)

State	Race	Δ (Slope)	95% Low	95% High
MO	Black	+7.87	+3.50	+12.23
MO	White	+7.94	+3.58	+12.30
SC	Black	+6.60	+2.24	+10.97

As denoted in Table 5 (Appendix B), using the first gamma log-link GLM with Wald tests to compare pre- and post-cutoff percentage growth in annual incidence rates among women born from 1944 to 1950 for sensitivity analysis, I again observed a significant decline in percentage growth of incidence per 100,000 among non-Hispanic Black women in Missouri from the pre-cutoff period to the post-cutoff period. Additionally, I once again found the post-cutoff percentage growth rate in this cohort to be significantly lower than the corresponding percentage growth rates observed in both non-Hispanic White women in Missouri (*p* = 0.001) and non-Hispanic Black women in South Carolina (*p* = 0.026).

As denoted in Table 6 (Appendix B), using the second gamma log-link GLM to compare post-cutoff percentage growth in annual incidence rates among women born from 1937 to 1943, I again observed similar percentage growth in incidence per 100,000 among non-Hispanic Black and White women in Missouri and non-Hispanic Black women in South Carolina. Subsequent Wald testing once again confirmed that the percentage growth rate past the median age of 62 was not significantly lower among non-Hispanic Black Missouri women relative to either non-Hispanic White Missouri women (*p* = 0.246) or non-Hispanic Black South Carolina women (*p* = 0.267). Wald testing also confirmed that the percentage growth rate past the median age of 69 was not significantly lower among non-Hispanic Black Missouri women relative to non-Hispanic White Missouri women (*p* = 0.145) and non-Hispanic Black South Carolina women (p = 0.399) belonging to this birth cohort. Finally, as shown in Table 7 (Appendix B), I found through supplemental analysis of BRFSS data that screening rates within the 1944–1950 birth cohort consistently increased from 2008 to 2016 in both non-Hispanic Black and White women.

## Discussion

My serial segmented regression analyses of USCS annual breast cancer incidence data indicate that, despite increases in breast cancer screening from 2008 to 2016, non-Hispanic Black women in Missouri born between 1944 and 1950—who were 4–11 years of age at the time of Missouri public school integration in September 1955—saw significantly reduced growth in breast cancer incidence from a median age of 62 (in 2009) onward relative to non-Hispanic White women in Missouri and non-Hispanic Black women in South Carolina—where public schools would remain segregated for another 15 years—belonging to the same birth cohort. I observed no such divergences in growth among non-Hispanic Black and White women in Missouri and South Carolina born from 1937 to 1943 past either a median age of 62 (in 2002) or 69 (in 2009), implying that the divergence observed in non-Hispanic Black Missouri women belonging to the younger birth cohort is likely attributable to cohort-specific effects rather than age effects or contemporaneous period effects impacting both birth cohorts around 2009 (i.e., improved access to breast cancer screenings and/or routine physical exams). My findings suggest that providing students with access to better-quality education before adolescence—during which literacy and numeracy become considerably less malleable—may be associated with protective spillover effects later in life as they reach higher-risk ages for breast cancer. In congruence with Goldman and Smith, I propose two mechanisms for why better-quality education might matter: first, a better-quality education enhances general literacy and therefore supports greater health literacy (the ability to obtain and process health-related information), and second, it also improves individuals’ capacity to comprehend their physicians’ guidance for preventing and/or managing chronic diseases [6]. In the case of breast cancer detection, both mechanisms may play key roles in an individual’s decision to screen regularly and/or start screening at an earlier age.

Possible threats to the validity of these findings primarily arise from the fact that the Affordable Care Act (ACA) was enacted at approximately the same time the younger birth cohort reached a median age of 62. However, as the major provisions of the ACA aimed to expand health insurance coverage among working-class adults below the age of 65, particularly those with pre-existing conditions, passage of the ACA would only explain why non-Hispanic Black women in Missouri born between 1944 and 1950 saw substantial reductions in breast cancer incidence if the statute were observed to have had inverse consequences on uninsurance within the state, as uninsurance limits access to preventive screenings [19]. Given that data from the U.S. Census Bureau shows that the uninsurance rate fell by 4.0 percentage points in Missouri (13.9% to 8.9%) and 10.5 percentage points in South Carolina (20.5% to 10.0%) from 2010 to 2016, I dismiss the ACA’s passage as a likely alternative explanation for this key finding [20].

That said, I acknowledge this analysis to have been constrained by the chosen dataset, as using state-level incidence data limited my ability to directly account for individual differences in socioeconomic status, health behaviors, and access to care that may have resulted in varying breast cancer risk and diagnosis timing; I additionally did not have true group population counts available for most of the study period (with the exception of 2010) to estimate annual incidence per 100,000 from. The SEER program’s evolving coding and staging guidelines over time also introduced potential bias—though I would expect such changes to have similarly affected year-to-year reported incidence in both non-Hispanic Black and White women. Lastly, although Federal Reserve data showed that ~70% of individuals born in Missouri and South Carolina continue to reside in their respective states of birth throughout their lifetimes, the nature of the chosen dataset made it impossible to ascertain what percentages of the reported cases and estimated populations were actually exposed to state-specific school integration policies, leaving the study’s findings susceptible to potential exposure misclassification bias due to migration to/from the states of interest. It is possible that, given access to better-quality education earlier on in their lifetimes, members of the 1944–1950 non-Hispanic Black birth cohort in Missouri may have migrated out of the state in pursuit of higher education and/or employment opportunities at a greater rate than their predecessors, potentially contributing to the underestimation of the true magnitude of reduction in annual breast cancer incidence associated with exposure to school integration.

Overall, given the stated limitations, future research leveraging individual-level datasets such as SEER-Medicare data would be invaluable in clarifying causal mechanisms through which better-quality education influences cancer risk.

### Policy implications

While *de jure* segregation has long since been abolished in all 50 states, de facto public school segregation still persists today, even in non-historically Jim Crow states, due to school assignment based on home addresses [21]. New Jersey, for example, ranks among one of the most segregated states in the nation in terms of interaction between Black and White public school students, surpassing all 20 historically Jim Crow states [22]. As a result of *Abbott v. Burke,* a 1985 civil case where the Superior Court of New Jersey ruled that public school education in disadvantaged communities throughout the state was unconstitutionally substandard, the New Jersey School Development Authority currently oversees the operations of 31 underperforming “SDA” school districts, 21 of which were predominantly Black and/or Hispanic as of 2010 [23]. Despite receiving 60% of New Jersey’s education aid while comprising just 5% of all municipal public school districts in the state, average mathematics and language arts proficiency rates in SDA districts have consistently remained 25 and 30 percentage points lower, respectively, than in non-SDA districts [24, 25]. The New Jersey State Commissioner of Education’s 2012 Funding Report also notes that only 14% of Black and 21% of Hispanic students in New Jersey met the SAT’s College-Readiness Benchmark in 2011, compared to 51% of White students [23]. My findings suggest that if policymakers in New Jersey were to overhaul the state’s current SDA system by consolidating SDA school districts with non-SDA school districts in neighboring municipalities to combat de facto school segregation, whether through more equitable school attendance boundaries or centralized school choice lotteries, younger students currently assigned to SDA districts might eventually benefit not only from improved core subject proficiency and college readiness but also later in life from associations between better-quality education and reduced incidence of breast cancer and other chronic diseases.

## Data Availability

The data in the U.S. Cancer Statistics public use database come from the CDC’s National Program of Cancer Registries (NPCR) and the National Cancer Institute’s Surveillance, Epidemiology, and End Results (SEER) Program. Instructions for obtaining access to the U.S. Cancer Statistics database can be found at: https://www.cdc.gov/united-states-cancer-statistics/public-use/access-data.html

## Appendix

See Tables 5, 6, 7, and 8

### Appendix A—Statistical analysis

The piecewise regression model used to compare incidence among non-Hispanic Black and White women in Missouri and South Carolina born from 1944 to 1950 was as follows:

$$\hat{Y}_{t} \, = \,\beta_{0} \, + \,\beta_{1} *median \, age_{t} \, + \,\beta_{j} *group_{j} \, + \,\beta_{j + 3} *\left( {group_{j} *median \, age_{t} } \right)\, + \,\beta_{8} *age \, spline_{t} \, + \,\beta_{j + 7} * \left( {group_{j} *age \, spline_{t} } \right)\, + \,\varepsilon$$

where *Ŷ*_*t*_ indicated predicted growth in a cohort’s breast cancer incidence rate per 100,000 at timepoint t, *median age*_*t*_ indicated the median age of the cohort of interest at timepoint t, *group*_*j*_ indicated the specific demographic of interest (non-Hispanic Black or White women in either Missouri or South Carolina), and *age spline*_*t*_ indicated the number of years at timepoint t since the cutoff point at 62 (the median age of breast cancer diagnosis). *ꞵ*_*0*_ and *ꞵ*_*1*_ estimated base level breast cancer incidence rates in 2001 and baseline growth in annual incidence rates, while *ꞵ*_*2*_ through* ꞵ*_*4*_ estimated group effects for non-Hispanic White women in Missouri, non-Hispanic Black women in South Carolina, and non-Hispanic White women in South Carolina, respectively (with non-Hispanic Black women in Missouri serving as the reference group). *ꞵ*_*5*_ through* ꞵ*_*7*_ estimated interactions between group and median age, while *ꞵ*_*8*_ estimated the additional effect of reaching the median age of breast cancer diagnosis on annual incidence rates. Finally, *ꞵ*_*9*_ through* ꞵ*_*11*_ estimated interactions between group and the age spline, while *ε* estimated residual error.

Additionally, the adjusted piecewise regression model used to compare incidence among the whole dataset (non-Hispanic Black and White women in Missouri and South Carolina born from either 1937 to1943 or 1944 to 1950) was as follows:

$$\hat{Y}_{t} \, = \,\beta_{0\,} \, + \,\beta_{1} *median\,age_{t} \, + \,\beta_{j} *group_{j} \, + \,\beta_{j + 7} *\left( {group_{j} *median\,age_{t} } \right)\, + \,\beta_{16} *age\,spline_{t} + \beta_{j + 15} * \left( {group_{j} *age\,spline_{t} } \right) + \gamma_{year} *D_{year} + \varepsilon$$

with *group*_*j*_ now indicating race, state, and birth cohort, while the definitions of *Ŷ*_*t*_, *ꞵ*_*0*_, *ꞵ*_*1*_, *median age*_*t*_, *age spline*_*t*_*, and ε* remained unchanged.* ꞵ*_*2*_ through *ꞵ*_*8*_ estimated group effects for non-Hispanic Black women in Missouri born from 1937 to 1943, non-Hispanic White women in Missouri born from 1944 to 1950, non-Hispanic White women in Missouri born from 1937 to 1943, non-Hispanic Black women in South Carolina born from 1944 to 1950, non-Hispanic Black women in South Carolina born from 1937 to 1943, non-Hispanic White women in South Carolina born from 1944 to 1950, and non-Hispanic White women in South Carolina born from 1937 to 1943, respectively (with non-Hispanic Black women in Missouri born from 1944 to 1950 serving as the reference group), while *ꞵ*_*9*_ through *ꞵ*_*15*_ estimated interactions between group and median age. *ꞵ*_*16*_ estimated the additional effect of reaching the median age of breast cancer diagnosis on annual incidence rates, while *ꞵ*_*17*_ through *ꞵ*_*23*_ estimated interactions between group and the age spline. Finally, *D*_*year*_ served as a dummy variable for year of observation, while *γ*_*year*_ estimated the period effect.

The first of my two generalized linear models, again used to compare growth in incidence rates across various demographics born from 1944 to 1950, was as follows:

$$\log \left( {E\left[ {\hat{Y}_{t} } \right]} \right)\, = \,\beta_{0} \, + \,\beta_{1} *median \, age_{t} \, + \,\beta_{j} *group_{j} \, + \,\beta_{j + 3} *\left( {group_{j} *median \, age_{t} } \right)\, + \,\beta_{8} *age \, spline_{t} \, + \,\beta_{j + 7} * (group_{j} *age \, spline_{t} )$$

where *log(E[Ŷ*_*t*_*])* estimated annual predicted percentage growth in breast cancer incidence rate per 100,000 given *median age*_*t*_, *group*_*j*_, *and age spline*_*t*_. Definitions for all inputs and coefficients on the right-hand side of the equation remained unchanged from the corresponding piecewise regression model. The second model, used to compare growth in incidence rates among the whole dataset, was as follows:

$$\log \left( {E\left[ {\hat{Y}_{t} } \right]} \right)\, = \,\beta_{0} \, + \,\beta_{1} *median \, age_{t} \, + \,\beta_{j} *group_{j} \, + \,\beta_{j + 7} *\left( {group_{j} *median \, age_{t} } \right)\, + \,\beta_{16} *age \, spline\, + \,\beta_{j + 15} * \left( {group_{j} *age \, spline_{t} } \right)\, + \,\gamma_{year} *D_{year}$$

where *log(E[Ŷ*_*t*_*])* is the estimated annual predicted percentage growth in the breast cancer incidence rate per 100,000 given *median age*_*t*_, *group*_*j*_, *age spline*_*t*_, and *year*. Once again, definitions for all inputs and coefficients on the right-hand side of the equation remained unchanged from the corresponding piecewise model.

### Appendix B—Sensitivity/robustness

**Table 5:** Pre- and post-cutoff percentage growth in incidence per 100,000 (1944–1950 births)

		Pre-cutoff (median ages 54–62)			Post-cutoff (median ages 62–72)		
State	Race	Δ (%)	95% Low	95% High	Δ (%)	95% Low	95% High
MO	Black	+6.20	+4.22	+8.18	+1.25	-0.34	+2.84
MO	White	+4.11	+2.12	+6.10	+4.59	+3.02	+6.16
SC	Black	+4.83	+2.84	+6.82	+3.45	+1.90	+5.00

**Table 6:** Post-cutoff percentage growth in incidence per 100,000 (1937–1943 births)

State	Race	Δ (%)	95% Low	95% High
MO	Black	+2.07	+0.98	+3.17
MO	White	+1.66	+0.59	+2.73
SC	Black	+1.70	+0.63	+2.77

**Table 7:** Breast cancer screening rates by race and state

State	Race	Age (2008)	Screening rate	Age (2016)	Screening rate	P.P. change
MO	Black	55–64	83.3%	65–74	85.5%	+2.2%
MO	White	55–64	78.5%	65–74	79.1%	+0.6%
SC	Black	55–64	83.6%	65–74	85.1%	+1.5%
SC	White	55–64	78.5%	65–74	80.9%	+2.4%

### Appendix C—Population estimates

**Table 8:** Annual estimated group populations by state, birth cohort, and race (2000–2020)

*State*	Missouri (MO)				South Carolina (SC)			
*Cohort*	1944–1950 Births		1937–1943 Births		1944–1950 Births		1937–1943 Births	
*Race*	Black	White	Black	White	Black	White	Black	White
2000	22,922	206,525	16,145	159,767	46,470	128,799	30,880	99,412
2001	22,818	206,654	15,963	158,936	46,579	130,701	30,807	100,172
2002	22,711	206,743	15,778	158,065	46,678	132,579	30,726	100,898
2003	22,595	206,751	15,586	157,121	46,758	134,410	30,625	101,553
2004	22,467	206,640	15,385	156,063	46,809	136,170	30,496	102,104
2005	22,324	206,367	15,170	154,856	46,823	137,834	30,329	102,515
2006	22,161	205,894	14,939	153,461	46,788	139,380	30,115	102,751
2007	21,974	205,180	14,689	151,840	46,696	140,783	29,844	102,778
2008	21,760	204,185	14,417	149,957	46,537	142,020	29,508	102,561
2009	21,514	202,669	14,118	147,773	46,302	143,067	29,096	102,064
2010	21,232	201,191	13,790	145,252	45,981	143,900	28,599	101,253
2011	20,912	199,125	13,431	142,368	45,567	144,503	28,011	100,104
2012	20,557	196,698	13,042	139,146	45,068	144,893	27,338	98,641
2013	20,169	193,950	12,628	135,623	44,492	145,093	26,590	96,899
2014	19,755	190,921	12,191	131,838	43,849	145,126	25,776	94,912
2015	19,316	187,651	11,735	127,827	43,149	145,017	24,905	92,716
2016	18,858	184,180	11,262	123,629	42,401	144,789	23,987	90,345
2017	18,384	180,549	10,776	119,281	41,615	144,466	23,031	87,835
2018	17,898	176,797	10,281	114,820	40,800	144,072	22,047	85,219
2019	17,404	172,965	9,779	110,284	39,966	143,630	21,044	82,534
2020	16,906	169,093	9,274	105,710	39,123	143,165	20,032	79,814
